# Quercetin and Its Derivative Counteract Palmitate-Dependent Lipotoxicity by Inhibiting Oxidative Stress and Inflammation in Cardiomyocytes

**DOI:** 10.3390/ijerph20043492

**Published:** 2023-02-16

**Authors:** Maria Concetta Granieri, Carmine Rocca, Anna De Bartolo, Immacolata Cristina Nettore, Vittoria Rago, Naomi Romeo, Jessica Ceramella, Annaluisa Mariconda, Paolo Emidio Macchia, Paola Ungaro, Maria Stefania Sinicropi, Tommaso Angelone

**Affiliations:** 1 Laboratory of Cellular and Molecular Cardiovascular Pathophysiology, Department of Biology, Ecology and Earth Science (DiBEST), University of Calabria, 87036 Rende, Italy; 2 Dipartimento di Medicina Clinica e Chirurgia, Scuola di Medicina, Università degli Studi di Napoli Federico II, 80131 Naples, Italy; 3 Department of Pharmacy, Health and Nutritional Sciences, University of Calabria, 87036 Rende, Italy; 4 Department of Science, University of Basilicata, Viale dell’Ateneo Lucano 10, 85100 Potenza, Italy; 5 Istituto per l’Endocrinologia e l’Oncologia Sperimentale (IEOS) “Gaetano Salvatore”, Consiglio Nazionale delle Ricerche, 80131 Naples, Italy; 6 National Institute of Cardiovascular Research (INRC), 40126 Bologna, Italy

**Keywords:** cardiomyocytes, lipotoxicity, flavonoids, quercetin

## Abstract

Cardiac lipotoxicity plays an important role in the pathogenesis of obesity-related cardiovascular disease. The flavonoid quercetin (QUE), a nutraceutical compound that is abundant in the “Mediterranean diet”, has been shown to be a potential therapeutic agent in cardiac and metabolic diseases. Here, we investigated the beneficial role of QUE and its derivative Q2, which demonstrates improved bioavailability and chemical stability, in cardiac lipotoxicity. To this end, H9c2 cardiomyocytes were pre-treated with QUE or Q2 and then exposed to palmitate (PA) to recapitulate the cardiac lipotoxicity occurring in obesity. Our results showed that both QUE and Q2 significantly attenuated PA-dependent cell death, although QUE was effective at a lower concentration (50 nM) when compared with Q2 (250 nM). QUE decreased the release of lactate dehydrogenase (LDH), an important indicator of cytotoxicity, and the accumulation of intracellular lipid droplets triggered by PA. On the other hand, QUE protected cardiomyocytes from PA-induced oxidative stress by counteracting the formation of malondialdehyde (MDA) and protein carbonyl groups (which are indicators of lipid peroxidation and protein oxidation, respectively) and intracellular ROS generation, and by improving the enzymatic activities of catalase and superoxide dismutase (SOD). Pre-treatment with QUE also significantly attenuated the inflammatory response induced by PA by reducing the release of key proinflammatory cytokines (IL-1β and TNF-α). Similar to QUE, Q2 (250 nM) also significantly counteracted the PA-provoked increase in intracellular lipid droplets, LDH, and MDA, improving SOD activity and decreasing the release of IL-1β and TNF-α. These results suggest that QUE and Q2 could be considered potential therapeutics for the treatment of the cardiac lipotoxicity that occurs in obesity and metabolic diseases.

## 1. Introduction

Obesity is a multifactorial disease, characterized by a complex aetiology that includes genetic, environmental, and behavioural aspects [1,2]. Several preclinical and clinical studies suggest a critical association between obesity and cardiovascular diseases (CVDs), especially in the context of heart failure (HF) [1]. Accordingly, obesity is associated with increased cardiovascular risk and a predisposition to coronary heart disease, hypertension, and other clinical conditions including diabetes and insulin resistance [1,3]. Following the accumulation of lipids in various organs, a process known as lipotoxicity, there is an alteration in intracellular signalling, mitochondrial dysfunction, and apoptosis, leading to organ dysfunction [4]. Excess triglycerides and circulating free fatty acids (FFAs), especially palmitate (PA), are mainly responsible for lipotoxicity in the heart [5].

Although lipids are essential for membrane structure and β-oxidation and cardiomyocytes use FAs as the main fuel for energy production under physiological conditions [6], their excess and accumulation—which occurs when the cardiac uptake of FAs increases compared to the rate of FA oxidation—are harmful because they can activate inflammatory pathways that accelerate the progression of cellular damage and lead to contractile dysfunction and HF. On the other hand, PA oxidation delivers excess electrons to the ETC, leading to mitochondrial uncoupling and superoxide overproduction, which subsequently activates stress-sensitive signalling pathways and triggers oxidative stress [7,8,9]. However, there are conflicting results to indicate that the acceleration of β-oxidation can attenuate oxidative stress in excess FA, making cardiac lipotoxicity a complex and multifactorial process. In cardiac lipotoxicity, the accumulation of various metabolites, including ceramides, diacylglycerols, and PA, leads to cell death [10,11,12]. Accordingly, it has been suggested that elevated serum levels of PA, the major circulating saturated FA, are a possible factor in the development of lipotoxic cardiomyopathy [13]. On the other hand, PA contributes significantly to obesity-associated HF, mainly by inducing inflammatory damage to the myocardium through the activation of toll-like receptor 4 [14].

Several natural and synthetic compounds have been tested for their ability to selectively act on different signalling pathways to promote cardioprotection in experimental models of lipotoxic cardiomyopathy [15]. However, our knowledge of the molecular mechanisms underlying cardiac lipotoxicity needs further improvement to identify specific approaches to minimizing cardiac lipotoxicity and obesity-related cardiac complications.

In recent years, there has been a significant increase in interest in the biological effects of natural compounds and their beneficial role in various metabolic and cardiovascular diseases [16,17,18]. In this context, the flavonoid quercetin (QUE) (2-(3,4-dihydroxyphenyl)-3,5,7-trihydroxychromen-4-one), which belongs to the class of flavonols, is a nutraceutical compound abundant in the “Mediterranean diet”. QUE is present in various fruits and vegetables such as radishes, onions, and cranberries, and can also be found in tea and red wine. This flavonol has numerous properties, ranging from anticancer to anti-inflammatory, antihypertensive, and antioxidant effects [19,20]. For example, QUE is known to induce the degradation of superoxide anions and lipid peroxy radicals, activate key antioxidant enzymes, inhibit oxidases, and attenuate oxidative stress induced by nitric oxide [21]. Further studies in animal models have shown that QUE has anti-obesogenic, anti-hypercholesterolemic, and anti-adipogenic effects that counteract metabolic syndromes and cardiac dysfunction [22,23,24].

However, the therapeutic use of QUE still seems to be limited, mainly due to its poor stability in aqueous media and its poor bioavailability, which is due to the two phenolic hydroxyl groups in its chemical structure [25]. Therefore, several studies have been conducted to improve the pharmacokinetic properties and chemical stability of QUE. In this context, several QUE derivatives were identified in which all hydroxyl groups were replaced by hydrophobic functional groups. Among them, we previously developed Q2 (2-(3,4-Diacetoxyphenyl)-4-oxo-4H-chromene-3,5,7-triyl triacetate), in which all hydroxyl groups were replaced by acetyl groups, resulting in improved hydrophobicity and stability and an expected higher bioavailability when compared to QUE [19,25]. The derivative Q2 exhibited a better cytotoxic profile than QUE in two breast cancer models with no toxic effect on normal cells. It also demonstrated the ability to inhibit human topoisomerases [26]. Moreover, Q2 was able to reverse the unfavourable epigenomic profiles associated with the occurrence of obesity [19,25].

Based on this knowledge and given the biological properties of QUE in cardiac and metabolic pathophysiology, we tested the potential beneficial effects of QUE and Q2 against cardiac lipotoxicity in H9c2 cardiomyocytes exposed to PA as a model of lipid overload.

## 2. Materials and Methods

### 2.1. Chemicals and Reagents

Quercetin and its derivative, Q2, were chemically synthesized as previously reported in [25]. Sodium palmitate (PA), 3-(4,5-Dimethylthiazol-)2,5-diphenyl Tetrazolium Bromide (MTT), β-nicotinamide adenine dinucleotide (NADH), Na-pyruvate, 2-thiobarbituric acid (TBA), butanol-1, butylated hydroxyanisole, bovine serum albumin (BSA), ethylene-diaminetetraacetic acid (disodium salt), diethyl ether, and Tween-20 were purchased from Sigma Aldrich (Saint Louis, MO, USA). Dulbecco’s Modified Eagle Medium/Nutrient Mixture F-12 (DMEM/F-12), Dulbecco’s phosphate-buffered saline (DPBS), penicillin/streptomycin, 0.25% Trypsin-EDTA (1X), and foetal bovine serum (FBS) were purchased from Thermo Fisher Scientific (Waltham, MA, USA). Dimethyl sulfoxide (DMSO), bovine serum albumin (BSA), and non-fat dried milk were purchased from PanReac AppliChem (Glenview, IL, USA). Absolute ethanol, hydrochloric acid, ethyl acetate, trichloro-acetic acid, and methanol were obtained from Carlo Erba Reagents (Cornaredo, MI, Italy).

### 2.2. Cell Culture

H9c2 cardiomyocytes were purchased from the American Type Culture Collection (ATCC) (Manassas, VA, USA) (Cat# CRL -1446) and cultured as described in our previous publications [27,28,29,30]. When cell confluence reached 80%, H9c2 cardiomyocytes were digested in a 1:2 ratio with 0.25% trypsin-EDTA (1X) (Gibco), according to the manufacturer’s instructions (ATCC). For all experiments, H9c2 cells were plated in complete medium and incubated at 37 °C and 5% CO_2_ for 48 h, as previously reported [27,28,29,30].

### 2.3. Cell Viability Assay

The viability of the H9c2 cells was determined by the 3-(4,5-dimethylthiazol-)2,5-diphenyl tetrazonium bromide (MTT) assay as previously described [27,29,30]. In brief, H9c2 cardiomyocytes (5 × 10^3^ cells/well) were seeded in 96-well plates and then treated with palmitate (PA) (from 100 to 500 µM) or vehicle (BSA), indicated as a control, for 24 h. To prepare a 10 mM stock solution, PA (Sigma Aldrich) was solubilized with 10% BSA fatty-acid-free at a molar ratio of 6:1 (PA: BSA) [8]. Once the concentration for PA-induced cell injury was established, H9c2 cardiomyocytes were pre-treated with QUE (from 1 to 1000 nM) or Q2 (from 1 to 1000 nM) for 2 h [31] and then exposed to PA (200 μM) for 24 h. The baseline effects of both compounds (QUE and Q2) on cell viability were examined for 24 h at a concentration range of 1–1000 nM. Control cells were treated with the vehicle (0.2% BSA). After the treatments, the medium was replaced with 100 µL of a 2 mg/mL MTT solution (Sigma Aldrich). The cells were then incubated at 37 °C and 5% CO_2_ for 4 h. The MTT solution was then removed and DMSO was added to dissolve the formazan crystals. The absorbance was measured at 570 nm using a microplate reader (Multiskan™ SkyHigh, Thermo Fisher Scientific Inc.). The mean absorbance values of six wells in each experimental group were expressed as a percentage of cell viability compared to control cells. The experiment was independently repeated three times.

### 2.4. Lactate Dehydrogenase (LDH) Evaluation

Cell injury induced by treatment with PA in H9c2 cardiomyocytes was assessed by determining the LDH release in the culture medium. H9c2 cells (1 × 10^5^ cells/mL) were seeded in a 24-well plate, pre-treated with QUE (50 nM) or Q2 (250 nM) for 2 h, and then treated with PA (200 μM) for 24 h. After the treatments, 100 μL/well of culture medium was used for the spectrophotometric determination of LDH activity, using a Multiskan™ SkyHigh (Thermo Fisher Scientific) according to the McQueen method [32]. The reaction rate was evaluated by the decrease in absorbance at 340 nm as a result of NADH oxidation and as an indicator of LDH activity. Enzyme activity was expressed in IU/L [33].

### 2.5. Oil Red O Staining

Lipid accumulation in cardiomyocytes was measured by Oil Red O staining. For this purpose, H9c2 cells were seeded in 6-well plates, pre-treated with QUE or Q2 for 2 h, and then treated with PA (200 μM) for 24 h at 37 °C in a humidified atmosphere. After the treatments were completed, the cells were washed three times with DPBS and incubated with an Oil Red O kit according to the manufacturer’s instructions (#04-220923, Bio Optica, Milan, Italy). H9c2 cells were incubated with reagent A for 20 min and, after washing, incubated with reagent B for 30 s and distilled water for 3 min [34,35,36]. Cells were then visualized under an Olympus BX41 microscope, and images were acquired with CSV1.14 software. A CAM XC -30 was used for image acquisition.

### 2.6. Assessment of Malondialdehyde (MDA) Concentration

MDA concentration, an index of lipid peroxidation after the production of ROS, was determined by the thiobarbituric acid reactive substances assay (TBARS) as previously described [27,37,38]. In brief, H9c2 cardiomyocytes were seeded at a density of 1 × 10^5^ cells/mL, pre-treated with QUE or Q2, and then exposed to PA for 24 h, as described. At the end of the treatments, cells were washed with cold DPBS, centrifuged at 300× *g* for 10 min, and the supernatant was removed. The pellets obtained by centrifugation were resuspended in 1 mL of a solution of cold sodium phosphate buffer 10 mM (containing 1 mM ethylenediaminetetraacetic acid and 1 mM butylated hydroxyanisole in 0.15% ethanol). All samples were then crushed intermittently with ultrasonic waves. The MDA concentration was determined spectrophotometrically using a Multiskan™ SkyHigh (Thermo Fisher Scientific Inc.), and the absorbance difference (535–600 nm) was converted to MDA equivalents using the extinction coefficient for MDA, 1.55 × 10^5^ M^−1^ cm^−1^. Lipid peroxidation assessment in terms of MDA concentration was expressed in mmol/L.

### 2.7. Measurement of Protein Carbonyl Content

Protein carbonyl group content, an indicator of protein damage induced by oxidative stress, was determined by the 2,4-dinitrophenylhydrazine (DNPH) assay according to the method of Reznick and Packer [39] and as previously reported [27,40]. H9c2 cardiomyocytes were seeded at a density of 1 × 10^5^ cells/mL, pre-treated with QUE for 2 h, and then exposed to PA for 24 h as previously described. They were then washed with cold DPBS, centrifuged at 300× *g* for 10 min, and the supernatant was removed. The obtained pellets were resuspended in 1 mL of a cold phosphate buffer (50 mM; pH 6.7) containing 1 mM of ethylenediaminetetraacetic acid and sonicated. Protein carbonyl group content (expressed in μmol/L) was determined spectrophotometrically (Multiskan™ SkyHigh, Thermo Fisher Scientific Inc.) at 375 nm using the extinction coefficient for DNPH (22 mM^−1^ cm^−1^).

### 2.8. Evaluation of Superoxide Dismutase (SOD) Enzyme Activity

The enzymatic activity of SOD was evaluated by assessing the inhibition of pyrogallol autoxidation according to the method of Marklund S. and Marklund G. [41] and as previously reported [27]. In brief, H9c2 cells were seeded at a density of 1 × 10^5^ cells/mL, pre-treated with QUE or Q2 for 2 h, and exposed to PA for 24 h. Cells were then washed with cold DPBS, centrifuged at 300× *g* for 10 min, and the supernatant was removed. The obtained precipitate was then resuspended in a Tris buffer (50 mM; pH 8.2) containing 100 mM of ethylenediaminetetraacetic acid. The reaction was initiated by adding 8 mM of pyrogallol. SOD activity was then evaluated spectrophotometrically (Multiskan™ SkyHigh, Thermo Fisher Scientific Inc.) at 420 nm by assessing the autoxidation of pyrogallol for 5 min at room temperature. It was expressed as the percentage of inhibition of pyrogallol autoxidation.

### 2.9. Catalase (CAT) Activity Assay

CAT activity was determined according to the method of Aebi [42]. In brief, H9c2 cardiomyocytes were seeded at a density of 1 × 10^5^ cells/mL, pre-treated with QUE for 2 h, and exposed to PA for 24 h. After the treatments, cells were lysed in 20 mM Tris/HCl buffer (pH 7.5) containing 0.2% Triton X-100 and 0.5 mM PMSF. They were then sonicated on ice for 30 s. All samples were centrifuged at 3000 rpm for 15 min at 4 °C, and the supernatant was used for the CAT activity assay. Specifically, 100 µL of the cell lysate was added to 1.9 mL of 50 mM phosphate buffer (pH 7.0) to start the reaction, adding 1 mL of 30 mM H_2_O_2_. Catalase activity was measured at 240 nm using a Multiskan™ SkyHigh spectrophotometer (Thermo Fisher Scientific). One unit of catalase was defined as the amount of enzyme capable of degrading 1 μmol of H_2_O_2_ per minute at 25 °C. The specific activity was expressed as the µmol of H_2_O_2_ degraded per minute per mg of protein.

### 2.10. Detection of Intracellular Reactive Oxygen Species (ROS)

ROS generation was assessed with the fluorescent probe CM -H_2_DCFDA, (5-(and-6)-chloromethyl-2′,7′-dichlorodihydrofluorescein diacetate acetyl ester) (C6827, Thermo Fisher Scientific). H9c2 cells (1 × 10^5^ cells/well) were seeded on coverslips in 6-well plates, pre-treated with QUE for 2 h, and then exposed to PA for an additional 24 h. Following the experimental protocol, cells were washed with DPBS and incubated with 1 μM CM-H_2_DCFDA in phenol- and serum-free DMEM/F-12 medium at 37 °C for 10 min in the dark according to the manufacturer’s instructions. At the end of the incubation period, H9c2 cells were washed with DPBS, and the CM-H_2_DCFDA probe was inactivated by adding the complete medium for 30 min at 37 °C. The fluorescence was analysed using an Olimpus fluorescence microscope (20× objective).

### 2.11. ELISAs Assay for the Assessment of Pro-Inflammatory Cytokines

To detect the release of the pro-inflammatory cytokines interleukin-1β (IL-1β) and tumour necrosis factor-α (TNF-α) (Elabscience Biotechnology Inc. USA), H9c2 cardiomyocytes were seeded, pre-treated with QUE or Q2 for 2 h, and exposed to PA for 24 h. After the treatments, the cell culture supernatant was collected, and analyses were performed by the ELISA assay according to the manufacturer’s instructions.

### 2.12. Statistical Analysis

Data were expressed as means ± SEM. Regarding the analyses, the one-way ANOVA test, followed by Dunnett’s multiple comparison test, and the Newman–Keuls multiple comparison test (for post-ANOVA comparisons) were used when appropriate. Values of * *p* <0.05, ** *p* <0.01, *** *p* <0.001, and **** *p* <0.0001 were considered statistically significant. Lines denote the comparisons among the experimental groups. The statistical analysis was conducted using Prism 5 (GraphPad Software, La Jolla, CA, USA).

## 3. Results

### 3.1. Effect of QUE and Its Derivative, Q2, on Cell Viability of H9c2 Cardiomyocytes Exposed to PA

**Figure 1:**
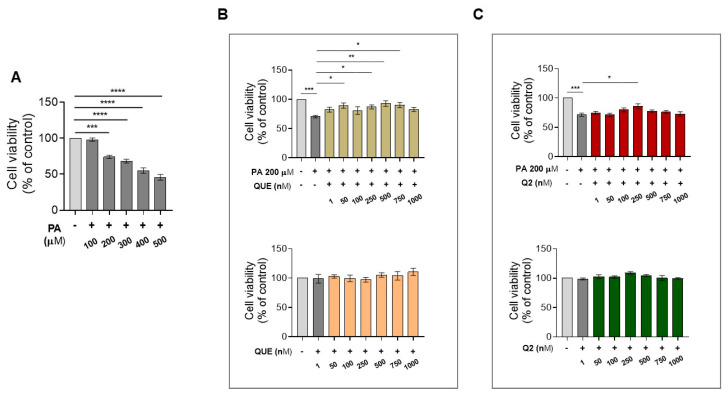
Effects of PA with or without QUE and Q2 on cell viability in H9c2 cardiomyocytes. H9c2 cardiomyocytes treated with vehicle (control) or increasing concentrations of (**A**) PA (100–500 µM) for 24 h; (**B**) QUE (1–1000 nM) for 2 h and PA 200 µM for 24 h or QUE alone (1–1000 nM) for 24 h; or (**C**) Q2 (1–1000 nM) for 2 h and PA 200 µM for 24 h or Q2 alone (1–1000 nM) for 24 h. Cell viability was determined by MTT assay and expressed as a percentage of control cells exposed to vehicle alone (indicated as Control). Results are represented as mean ± SEM (*n* = 6 per group). Significant differences (* *p* < 0.05, ** *p* < 0.01, *** *p* < 0.001, **** *p* < 0.0001) were detected by a one-way ANOVA, followed by Dunnett’s test vs. the control group and Newman–Keuls multiple comparison test vs. control group and vs. PA group.

To determine the concentration of PA that is capable of inducing a cytotoxic effect on cardiomyocytes, H9c2 cells were exposed to increasing concentrations of PA (100–500 μM) for 24 h. An assessment of cell viability using an MTT assay showed that PA caused a significant decrease in cell viability in a dose-dependent manner beginning at 200 μM compared with the control cells; therefore, this concentration was used for the subsequent experiments (Figure 1A). After determining the initial cytotoxic dose of PA in the in vitro model, we investigated the effect of QUE and its derived Q2 against PA-induced lipotoxic damage. For this purpose, H9c2 cardiomyocytes were pre-treated with increasing concentrations of both compounds, starting with 1 nM to 1000 nM for 2 h, followed by exposure to PA for 24 h. Our data showed that PA induced a significant decrease in cell viability compared with the control cells, whereas pre-treatment with QUE significantly protected the cardiomyocytes from PA-induced cell death at 50 nM, 250 nM, 500 nM, and 750 nM (Figure 1B). Therefore, the first effective concentration of QUE (50 nM) was used for subsequent experiments. The graph in Figure 1B shows that treatment with QUE alone, in the concentration range of 1–1000 nM, did not significantly affect cell viability when compared to control cells. On the other hand, in H9c2 cells pre-treated with Q2 (1–1000 nM) and then exposed to PA, the protective effect against PA-induced cell death was significant only at 250 nM (Figure 1C). Again, no significant effects were observed on the cell viability of H9c2 cells exposed to Q2 in the 1–1000 nM concentration range when compared with control cells (Figure 1C).

### 3.2. Action of QUE against PA-Dependent Cytotoxic and Lipotoxic Damage in Cardiomyocytes

**Figure 2:**
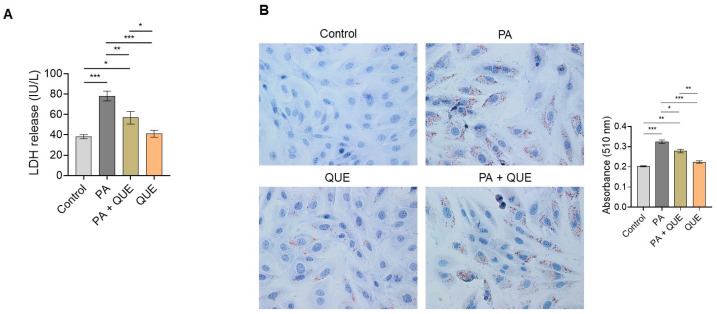
Effects of QUE on PA-induced cytotoxicity and lipid accumulation in H9c2 cardiomyocytes. (**A**) Lactate dehydrogenase (LDH) release in the culture medium of H9c2 cardiomyocytes treated with vehicle (control), PA (200 µM), and QUE (50 nM) for 2 h and then PA for additional 24 h or QUE alone (50 nM) for 24 h. LDH activity was expressed as IU/L and data represent the mean ± SEM of six separate experiments. Significant differences were detected by one-way ANOVA and Newman–Keuls multiple comparison test, *p* < 0.05 (*); *p* < 0.01 (**); and *p* < 0.001 (***). (**B**) Representative images of Oil Red O staining for intracellular lipid droplet assessment in H9c2 cardiomyocytes treated with vehicle (control), PA (200 µM), and QUE (50 nM) for 2 h and then PA for 24 h or QUE alone (50 nM) for 24 h. Quantification of the lipid droplets was performed by measuring the absorbance at 510 nm. Values are the mean ± SEM of three different experiments. Significant differences were detected by one-way ANOVA and Newman–Keuls multiple comparison test, *p* < 0.05 (*); *p* < 0.01 (**); and *p* < 0.001 (***). Scale bar: 25 µm.

To evaluate the effect of QUE against PA-induced cytotoxicity, the release of LDH in the culture medium was examined as a specific indicator of impaired structural membrane integrity after cell injury. As is shown in Figure 2A, treatment with PA significantly increased LDH release when compared with control cells, whereas pre-treatment with QUE caused a significant decrease in LDH activity when compared with H9c2 cells treated with PA alone. No significant changes in LDH levels were detected in the cells treated with QUE alone compared with control cells (Figure 2A). In addition, the assessment of intracellular lipid accumulation by Oil Red O staining initially showed an increase in intracellular lipids in H9c2 cells exposed to PA when compared with control cells; conversely, a significant decrease in lipid deposition was observed in the H9c2 cardiomyocytes pre-treated with QUE and then exposed to PA when compared with the PA group alone (Figure 2B).

### 3.3. Effects of QUE on PA-Provoked Oxidative Stress and Impaired SOD and CAT Enzymatic Activities in H9c2 Cardiomyocytes

**Figure 3:**
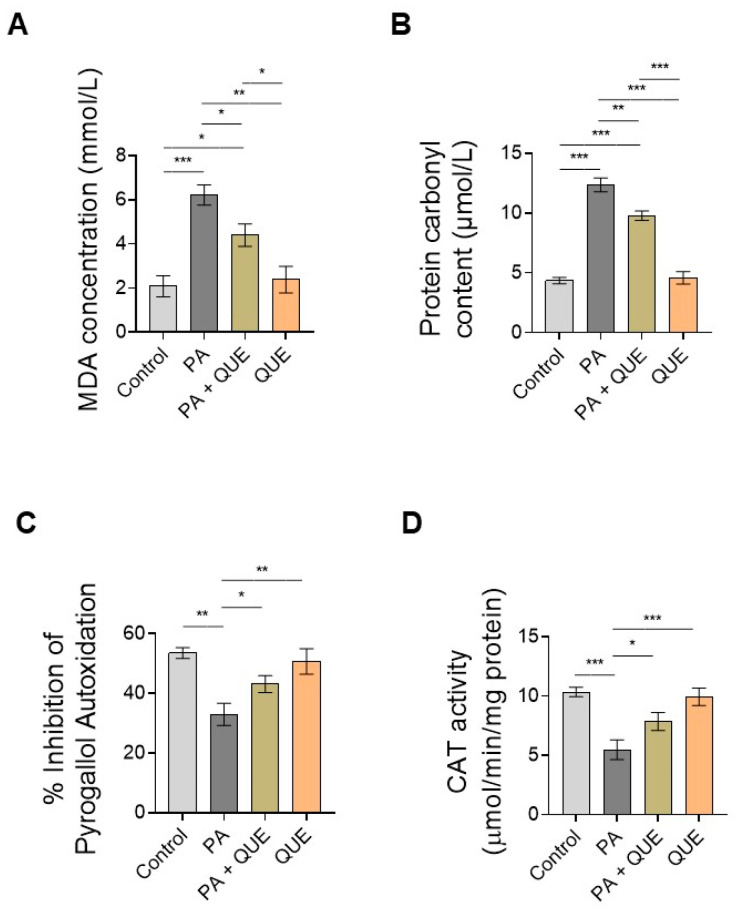
Effects of QUE on the levels of malondialdehyde (MDA) and protein carbonyls and the activities of superoxide dismutase (SOD) and catalase (CAT) in PA-treated cells. (**A**) MDA concentration, (**B**) protein carbonyl content, (**C**) SOD activity, and (**D**) CAT activity in lysates of H9c2 cardiomyocytes treated with vehicle (control), PA (200 µM), QUE (50 nM) for 2 h and then PA for 24 h, and QUE alone (50 nM) for 24 h. MDA concentration was expressed as mmol/L, the content of protein carbonyl groups was expressed as μmol/L, SOD activity was expressed as percentage of inhibition of the pyrogallol autoxidation rate, and CAT activity was expressed as μmol/min/mg protein. Data are expressed as the mean ± SEM of six separate experiments. Significant differences were detected by one-way ANOVA and Newman–Keuls multiple comparison test, *p* < 0.05 (*); *p* < 0.01 (**); and *p* < 0.001 (***).

To investigate whether pre-treatment with QUE can counteract PA-induced redox imbalance and oxidative stress, we first determined specific markers of oxidative stress (MDA and protein carbonyl content) and then measured the enzymatic activity of the key antioxidant enzymes SOD and CAT. Our results showed a significant increase in both oxidative-stress-associated markers in PA-treated cells when compared with the control cells with respect to MDA production (Figure 3A) and protein carbonyl groups (Figure 3B) (i.e., indicators of lipid peroxidation and protein damage after the generation of ROS). In contrast, a significant decrease in both indices was observed in the cardiomyocytes pre-treated with QUE that were subsequently exposed to PA when compared with the group exposed to PA alone (Figure 3A,B). On the other hand, both the percentage of pyrogallol autoxidation (an index of SOD activity) and CAT activity were significantly decreased in the PA-exposed H9c2 cells when compared with the control group. Both SOD and CAT levels were significantly increased in the cardiomyocytes that were pre-treated with QUE and exposed to PA when compared with the cells exposed to PA alone (Figure 3C,D). In contrast, treatment with QUE alone had no significant effects on the above parameters when compared with control cells (Figure 3A–D).

**Figure 4:**
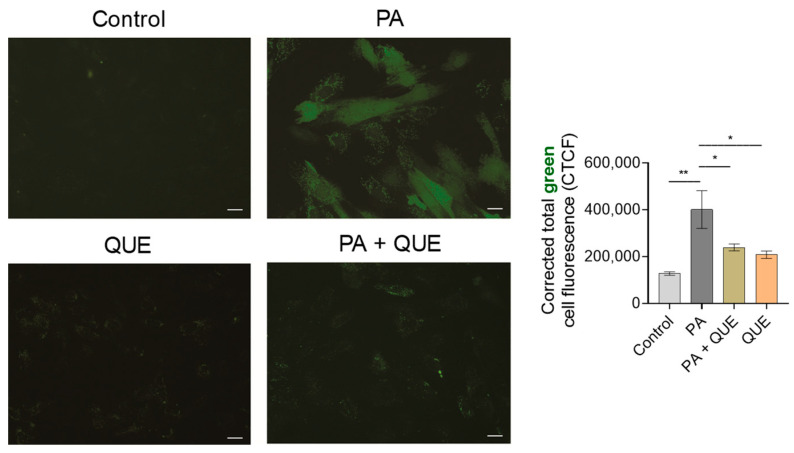
Effect of QUE pre-treatment on PA-dependent ROS overproduction in H9c2 cardiomyocytes. Representative images of a cell-permeable fluorescent probe (CM-H_2_DCFDA) for total ROS detection with relative fluorescence quantification. Data are expressed as the mean ± SEM (*n* = 2 different experiments). Significant differences in corrected total cell fluorescence (CTCF) were detected by one-way ANOVA and Newman–Keuls multiple comparison test, *p* < 0.05 (*) and *p* < 0.01 (**). Scale bar: 25 µm.

To confirm the antioxidant activity of QUE during PA exposure in cardiomyocytes, we also examined the intracellular ROS formation using the specific fluorescent probe CM-H_2_DCFDA. Our results showed that PA induced a significant increase in ROS production, evidenced by the significant increase in green fluorescence intensity compared with the control group, whereas pre-treatment with QUE in PA-exposed cardiomyocytes significantly decreased the fluorescence intensity when compared with the group that was treated with PA alone (Figure 4). Treatment with QUE alone had no significant effect on fluorescence intensity when compared with H9c2 cells exposed to vehicle (Figure 4).

### 3.4. Effect of QUE against PA-Dependent Inflammation in Cardiomyocytes

**Figure 5:**
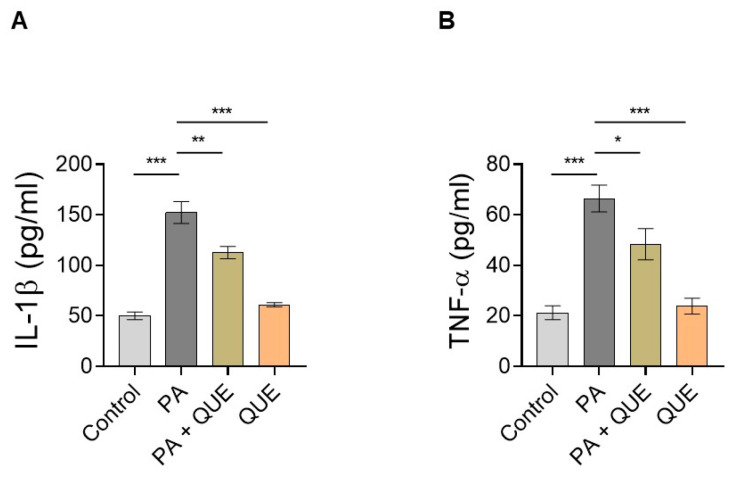
Effect of QUE against the release of PA-induced, pro-inflammatory cytokines in H9c2 cells. (**A**) IL-1β and (**B**) TNF-α levels in culture supernatant of H9c2 cardiomyocytes treated with vehicle (control), PA (200 µM), QUE (50 nM) for 2 h and then PA for 24 h, and QUE alone (50 nM) for 24 h. The levels of IL-1β and TNF-α, as specific pro-inflammatory markers, were expressed as pg/mL. Data are reported as the mean ± SEM of six separate experiments. Significant differences were detected by one-way ANOVA and Newman–Keuls multiple comparison test, *p* < 0.05 (*); *p* < 0.01 (**); *p* < 0.001 (***).

To evaluate the potential ability of QUE to counteract the PA-dependent inflammatory response, we focused on specific inflammatory markers by examining the release of the pro-inflammatory cytokines IL-1β and TNFα in the cell supernatant. The results showed that in the medium of H9c2 cells treated with PA, the levels of both IL-1β and TNFα were significantly increased when compared with control cells (Figure 5). In contrast, pre-treatment with QUE significantly reduced the release of these pro-inflammatory cytokines when compared with the PA group (Figure 5). Treatment with QUE alone did not result in significant changes in the inflammatory markers when compared with the control cells (Figure 5).

### 3.5. Effects of Q2 against PA-Provoked Cytotoxicity and Intracellular Lipid Accumulation in H9c2 Cardiomyocytes

**Figure 6:**
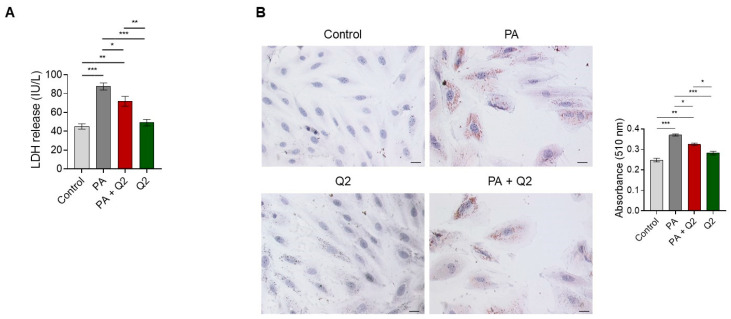
Effects of Q2 on PA-induced cytotoxicity and lipid accumulation in H9c2 cardiomyocytes. (**A**) Lactate dehydrogenase (LDH) release in the culture medium of H9c2 cardiomyocytes treated with vehicle (control), PA (200 µM), Q2 (250 nM) for 2 h and PA for additional 24 h, and Q2 alone (250 nM) for 24 h. LDH activity was expressed as IU/L and data represent the mean ± SEM of six separate experiments. Significant differences were detected by one-way ANOVA and Newman–Keuls multiple comparison test, *p* < 0.05 (*); *p* < 0.01 (**); and *p* < 0.001 (***). (**B**) Representative images of Oil Red O staining for intracellular lipid droplets assessment in H9c2 cardiomyocytes treated with vehicle (control), PA (200 µM), Q2 (250 nM) for 2 h and then PA for 24 h, and Q2 alone (250 nM) for 24 h. Quantification of the lipid droplets was performed by measuring the absorbance at 510 nm. Values are the mean ± SEM of three different experiments. Significant differences were detected by one-way ANOVA and Newman–Keuls multiple comparison test, *p* < 0.05 (*); *p* < 0.01 (**); and *p* < 0.001 (***). Scale bar: 25 µm.

In addition to QUE, we investigated the potential ability of Q2 to counteract lipotoxicity in H9c2 cardiomyocytes by testing the first effective concentration (i.e., 250 nM) at which Q2 significantly attenuated PA-provoked cell death (Figure 1C). Our results showed that in cardiomyocytes exposed to PA, the release of LDH in the culture medium was significantly increased when compared with control cells, whereas in cells pre-treated with Q2 and then exposed to PA, the release of LDH was significantly attenuated compared with cells treated with PA alone (Figure 6A). This analysis also showed that no significant changes in LDH levels were observed in the group treated with Q2 alone when compared with the control cells (Figure 6A). On the other hand, Oil Red O staining demonstrated a significant accumulation of intracellular lipids in the H9c2 cardiomyocytes treated with PA compared with the control cells, and a significant decrease in intracellular lipids in the PA and Q2 group compared with the PA-alone group (Figure 6B). Oil Red O staining also showed that there were no significant differences between the control group and the group treated with Q2 alone (Figure 6B).

### 3.6. Action of Q2 against PA-Dependent Oxidative Stress and Inflammation in H9c2 Cardiomyocytes

**Figure 7:**
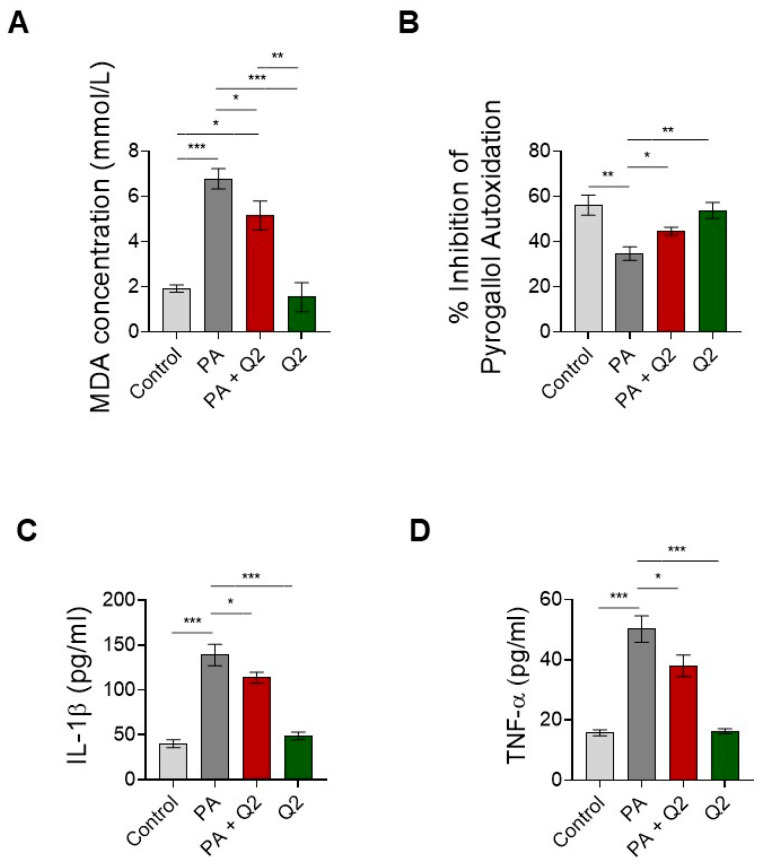
Effects of Q2 on the levels of malondialdehyde (MDA) and superoxide dismutase (SOD) activity and the levels of IL-1β and TNF-α in PA-treated cells. (**A**) MDA concentration, (**B**) SOD activity in lysates of H9c2 cardiomyocytes treated with vehicle (control), PA (200 µM), Q2 (250 nM) for 2 h and then PA for 24 h, and Q2 alone (250 nM) for 24 h. (**C**) IL-1β and (**D**) TNF-α levels in culture supernatant of H9c2 cardiomyocytes treated with vehicle (control), PA (200 µM), Q2 (250 nM) for 2 h and then PA for 24 h, and Q2 alone (250 nM) for 24 h. MDA concentration and SOD activity, as oxidative stress-related markers, were expressed as mmol/L and as percentage of inhibition of the pyrogallol autoxidation rate, respectively. The levels of IL-1β and TNF-α, as specific pro-inflammatory markers, were expressed as pg/mL. Data are expressed as the mean ± SEM of six separate experiments. Significant differences were detected by one-way ANOVA and Newman–Keuls multiple comparison test, *p* < 0.05 (*); *p* < 0.01 (**); and *p* < 0.001 (***).

To evaluate the ability of Q2 to counteract PA-induced oxidative stress and inflammatory response in cardiomyocytes, we first measured MDA and SOD concentrations and then the release of IL-1β and TNFα in the cell supernatant. Our data showed that MDA production increased significantly in the cells treated with PA compared with the control cardiomyocytes, whereas MDA production was significantly reduced in the cells that were pre-treated with Q2 and then exposed to PA when compared with the cells treated with PA alone (Figure 7A). In addition, the group treated with Q2 alone showed no significant change in MDA levels when compared with the control group (Figure 7A). An assessment of the enzymatic activity of SOD revealed an opposite trend, as it was significantly decreased in the cardiomyocytes treated with PA compared with the control group and significantly increased in the PA and Q2 group when compared with the PA alone group (Figure 7B). No significant changes in SOD levels were also detected in cells treated with Q2 alone compared with the control cells (Figure 7B). An analysis of the IL-1β and TNFα quantification in the supernatant of cardiomyocytes exposed to PA with or without Q2 showed a significant increase in both cytokines in the PA group when compared with the control group and a significant decrease in the PA and Q2 group when compared with the cells treated with PA alone (Figure 7C,D). In contrast, the levels of IL-1β and TNFα did not change significantly in the cardiomyocytes treated with Q2 alone compared with the control-treated cells (Figure 7C,D).

## 4. Discussion

It is well-known that the accumulation of fatty acids in non-adipose tissues, including cardiac and vascular tissues, can generate toxic intermediates, leading to cell death and lipotoxicity [43]. In particular, cardiac lipotoxicity, which occurs in metabolic diseases such as obesity, can lead to various CVDs, including diabetic cardiomyopathy and congestive heart failure [44,45,46,47]. Several complex mechanisms contributing to cardiac lipotoxicity and cellular dysfunction have been proposed. Although great efforts have been made to identify potentially effective strategies against cardiac lipotoxicity, a deeper understanding of the molecular relationships that drive lipotoxic, insult-dependent cardiac dysfunction remains necessary to characterize novel therapeutic approaches aimed at ameliorating cardiovascular complications secondary to obesity. In the present study, we used H9c2 cardiomyocytes exposed to PA, the major saturated FFA, to model cardiac lipotoxicity in vitro and to investigate the beneficial effects of the natural flavonoid QUE or its derivative, Q2.

### 4.1. QUE and Its Derivate, Q2, Counteract PA-Dependent Cardiomyocyte Death

There is ample evidence that elevated saturated FFAs, particularly PA, are responsible for lipotoxic damage to several cell types, including skeletal muscle cells, liver cells, neuronal cells/neuroblastoma cells, and cardiomyocytes [48,49,50] and references therein. Here, we exposed H9c2 cardiomyoblast cells—commonly used to recapitulate the main characteristics of primary cardiac cells in terms of their morphological, biochemical, and electrophysiological properties [51]—to PA to establish an in vitro model of hyperlipidaemia and to mimic the cardiac lipotoxicity that occurs in obesity.

Several literature reports suggest that PA induces myocardial damage that leads to cell death and cardiac dysfunction, thus providing an important method for inducing cardiac lipotoxicity in in vitro systems [52,53,54]. Accordingly, in this study, we first found that PA decreases cell viability and induces cell death in a dose-dependent manner (from 200 µM), indicating that an in vitro model of cytotoxicity induced by lipid overload has been successfully established. To date, numerous studies have been conducted on natural products and/or the chemical derivatives of natural compounds, as well as medicinal plant extracts, as novel agents for the treatment and/or prevention of cardiac lipotoxicity and cardiovascular and metabolic diseases due to their ability to act on specific signalling pathways involved in lipid metabolism, inflammation, energy production, and redox balance [15,16]. In addition to conventional oxidative-stress-related markers, recent evidence indicated the importance of emerging biomarkers, such as the balance of thiols, lipid peroxidation, protein carbonylation, and antioxidant barriers, which are increasingly recognized as supporting elements for the diagnosis/evaluation of metabolic dysfunction-related clinical manifestations and disease severity [55]. In this context, the use of phytochemicals (plant-derived small molecules) such as flavonoids could be advantageous as they have a higher pharmaceutical value than conventional drugs, thus circumventing the limitations of conventional pharmacological therapeutics. In fact, compared to conventional drugs, phytochemicals are less expensive and are readily available in natural foods, and their use may be particularly safe in long-term treatments [56,57]. Several preclinical data have identified some of the key mechanisms underlying the cardioprotective effects of phytochemical compounds, including QUE, a flavonoid abundant in the “Mediterranean diet”, in conjunction with their antioxidant, anti-inflammatory, and anti-apoptotic properties in various pathophysiological contexts [19,57,58,59,60,61,62,63] and references therein.

However, the potential beneficial effect of QUE on cardiac lipotoxicity has not yet been investigated. Here, we focused on a selective mechanism of hyperlipidaemia affecting the cardiomyocyte component. We found that the pre-treatment of cardiomyocytes with QUE during PA exposure resulted in increased cell viability, indicating the ability of QUE to attenuate PA-dependent cardiomyocyte death.

As QUE has relatively low bioavailability due to its poor water solubility and chemical stability, numerous studies have been conducted to develop selective QUE derivatives and to use formulation technologies to improve the pharmacokinetic properties and chemical stability of QUE [64,65,66]. To this end, we developed the penta-acetyl QUE derivative Q2, in which all OH groups were replaced by acetyl groups. We previously provided evidence that Q2 can improve the hydrophobicity and stability of QUE [65]. Interestingly, we found that Q2 also counteracted PA-dependent cardiomyocyte death, suggesting that Q2 is also effective against the cytotoxic effect of PA. However, in this study, we found that Q2 can attenuate PA-induced cell death at higher concentrations (i.e., 250 nM) than QUE (50 nM), suggesting a higher efficacy of QUE in combating PA-induced cytotoxicity at the cardiomyocyte level.

### 4.2. QUE Relieves PA-Induced Cytotoxicity and Accumulation of Lipid Droplets in Cardiomyocytes

The above results prompted us to further outline the mechanism of action of QUE during PA-induced lipotoxic damage in cardiomyocytes. We achieved this by first investigating whether QUE can reduce cytotoxicity and lipid-droplet accumulation as a result of PA exposure. Accordingly, evidence is accumulating that PA can disrupt physiological cellular signalling by promoting lipid deposition in cardiomyocytes, leading to cell death, increased LDH, and apoptosis [67,68]. Our results showed that QUE is able to reduce the PA-induced increase in LDH release in culture media, a specific marker of cytotoxicity and membrane damage, as well as intracellular lipid droplets [69], further confirming the protective effect of QUE against PA-induced lipotoxicity.

### 4.3. QUE Reduces PA-Induced Oxidative Stress and ROS Overproduction and Improves the Endogenous Antioxidant Defences

It is well-established that oxidative stress may be an important factor in the development and progression of obesity-related CVDs [70,71]. Both the overproduction of ROS and the impairment of endogenous antioxidant defence systems may trigger the activation of redox-sensitive signalling pathways that exacerbate the existing oxidative stress, caused by obesity per se, and drive the cardiovascular abnormalities induced by obesity [72]. Here, we reported that PA generates oxidative stress in cardiomyocytes by increasing MDA levels (i.e., a marker of lipid peroxidation), carbonylated proteins (an indicator of oxidative stress-dependent protein damage), and the intracellular production of ROS production. It and also negatively affects the activity of key endogenous antioxidant enzymes (SOD and CAT) required for the maintenance of cellular integrity against oxidative-stress-dependent damage and the repair of structural, oxidative, deleterious changes [73,74]. These data are consistent with numerous reports that the treatment of PA increases the production of ROS, resulting in lipid peroxidation, a reduction in antioxidant defences, and the perturbation of redox status toward an increasingly oxidative environment [67,75,76,77]. However, the pre-treatment of QUE in cardiomyocytes exposed to PA significantly decreased the intracellular formation of ROS, MDA content, and protein carbonylation. It also increased the enzymatic activities of SOD and CAT. These results suggest an intriguing potential of QUE to exert both direct and indirect antioxidant effects, thus preventing membrane damage resulting from the oxidation of FFAs, detoxifying the pro-oxidants that cause oxidative cell damage, and restoring intracellular redox status. Our results are also consistent with previous experimental evidence that highlighted the cardioprotective effects of QUE through antioxidant activity in mouse models of diabetes and obesity [78,79]. Considering that cardiomyocytes are particularly susceptible to oxidative damage because, compared to other organs such as the liver, they have relatively low levels of endogenous antioxidant systems, i.e., enzymes for the degradation of oxyradicals such as SOD and CAT [80,81], the ability of QUE to restore the activity of these important enzymes may be of particular pathophysiological interest.

### 4.4. QUE Mitigates PA-Provoked Inflammatory Response, Reducing the Release of Proinflammatory Cytokines

The hypothesis that lipotoxicity accelerates peripheral inflammation and that the FFAs secreted from visceral fat contribute to the activation of inflammation in cardiovascular and metabolic tissues is becoming increasingly clear [82]. Moreover, the accumulation of lipids triggers oxidative stress, leading to chronic inflammation and establishing a close relationship between these two molecular events, which inevitably affect myocardial structure and function, in cardiac lipotoxicity [83,84,85]. Specifically, ROS may promote inflammation in lipid overload by activating key signalling pathways involved in the release of proinflammatory cytokines, such as IL-1β and TNFα, which have deleterious effects on the heart [14,85,86,87,88]. The ability of QUE to attenuate the change in the redox status of cardiomyocytes and reduce intracellular ROS after PA prompted us to investigate whether QUE could also reduce the PA-dependent inflammatory response. Our results showed that the levels of pro-inflammatory cytokines TNFα and IL-1β in the cell medium were increased in PA-stimulated cardiomyocytes, which was also confirmed in previous studies [89,90,91], while administration of QUE reversed PA-induced inflammation, suggesting that it is able to exert a beneficial effect on cardiac lipotoxicity through an anti-inflammatory effect. These results are confirmed by previous studies, which found that QUE exerts a protective effect against atherosclerosis through its anti-inflammatory action and in obesity and insulin resistance by reducing inflammation and remodelling white adipose tissue, which may occur through inflammation-related mechanisms [92,93].

### 4.5. The QUE-Derivative Q2 Counteracts PA-Dependent Lipotoxicity by Mitigating Cytotoxicity, the Accumulation of Intracellular Lipids, Oxidative Stress, and Inflammation in Cardiomyocytes

The cytoprotective effect of QUE against lipotoxicity in cardiomyocytes raised the hypothesis that its selective derivative (i.e., Q2) might play a beneficial role under the same experimental conditions of lipotoxic injury. Therefore, we wondered whether the Q2 concentration capable of counteracting PA-induced cardiomyocyte death could also effectively antagonize the main mechanisms that drive PA lipotoxicity in cardiomyocytes. Interestingly, our analysis showed that Q2 exposure decreased the PA-induced release of LDH and intracellular lipid accumulation and also attenuated the effect of lipotoxicity on oxidative stress generation (i.e., MDA production and decreased SOD activity) and inflammatory response (i.e., the release of the pro-inflammatory cytokines TNFα and IL-1β). Our results, which further confirmed the biological activity of Q2 and suggest a protective role of this QUE derivative under lipotoxic conditions, are consistent with our previous data, which reported the ability of Q2 (and QUE) to counteract the unfavourable epigenomic profiles associated with obesity in three T3-L1 preadipocytes and in a rat model of obesity and metabolic syndrome [19] and also demonstrated enhanced cardiovascular activity in a rat model [25]. Although our data suggest that Q2 is effective against lipotoxic damage at higher concentrations when compared with QUE, it should be noted that, on the other hand, Q2 demonstrates better hydrophobicity, stability, and higher bioavailability than QUE; therefore, these results could be of potential interest for improving the therapeutic potential of QUE, which remains limited due to its poor bioavailability.

## 5. Conclusions

Overall, the results of the present study provide new evidence for the beneficial role of QUE in lipid overload in cardiomyocytes. Specifically, our study showed that QUE and its selective derivative (i.e., Q2, which demonstrates improved pharmacokinetic properties and chemical stability) can attenuate PA-dependent lipotoxicity by counteracting intracellular lipid accumulation and cell death, attenuating the inflammatory response and oxidative stress. Thus, QUE and Q2 can be considered potential therapeutics for the treatment of cardiac lipotoxicity that occurs in obesity and metabolic diseases.

## Data Availability

Data are contained within the article.

## References

[1] Ren J., Wu N.N., Wang S., Sowers J.R., Zhang Y. (2021). Obesity cardiomyopathy: Evidence, mechanisms, and therapeutic implications. Physiol. Rev..

[2] Gadde K.M., Martin C.K., Berthoud H.R., Heymsfield S.B. (2018). Obesity: Pathophysiology and Management. J. Am. Coll. Cardiol..

[3] Zhang Y., Sowers J.R., Ren J. (2012). Pathophysiological insights into cardiovascular health in metabolic syndrome. Exp. Diabetes Res..

[4] Unger R.H., Orci L. (2002). Lipoapoptosis: Its mechanism and its diseases. Biochim. Biophys. Acta.

[5] McGavock J.M., Lingvay I., Zib I., Tillery T., Salas N., Unger R., Levine B.D., Raskin P., Victor R.G., Szczepaniak L.S. (2007). Cardiac steatosis in diabetes mellitus: A 1H-magnetic resonance spectroscopy study. Circulation.

[6] Pasqua T., Rocca C., Giglio A., Angelone T. (2021). Cardiometabolism as an Interlocking Puzzle between the Healthy and Diseased Heart: New Frontiers in Therapeutic Applications. J. Clin. Med..

[7] Sletten A.C., Peterson L.R., Schaffer J.E. (2018). Manifestations and mechanisms of myocardial lipotoxicity in obesity. J. Intern. Med..

[8] Joseph L.C., Barca E., Subramanyam P., Komrowski M., Pajvani U., Colecraft H.M., Hirano M., Morrow J.P. (2016). Inhibition of NAPDH Oxidase 2 (NOX2) Prevents Oxidative Stress and Mitochondrial Abnormalities Caused by Saturated Fat in Cardiomyocytes. PLoS ONE.

[9] Ly L.D., Xu S., Choi S.K., Ha C.M., Thoudam T., Cha S.K., Wiederkehr A., Wollheim C.B., Lee I.K., Park K.S. (2017). Oxidative stress and calcium dysregulation by palmitate in type 2 diabetes. Exp. Mol. Med..

[10] Park M., Sabetski A., Kwan Chan Y., Turdi S., Sweeney G. (2015). Palmitate induces ER stress and autophagy in H9c2 cells: Implications for apoptosis and adiponectin resistance. J. Cell Physiol..

[11] Kim J.K., Fillmore J.J., Sunshine M.J., Albrecht B., Higashimori T., Kim D.W., Liu Z.X., Soos T.J., Cline G.W., O’Brien W.R. (2004). PKC-theta knockout mice are protected from fat-induced insulin resistance. J. Clin. Investig..

[12] Drosatos K., Bharadwaj K.G., Lymperopoulos A., Ikeda S., Khan R., Hu Y., Agarwal R., Yu S., Jiang H., Steinberg S.F. (2011). Cardiomyocyte lipids impair β-adrenergic receptor function via PKC activation. Am. J. Physiol. Endocrinol. Metab..

[13] Listenberger L.L., Han X., Lewis S.E., Cases S., Farese R.V., Ory D.S., Schaffer J.E. (2003). Triglyceride accumulation protects against fatty acid-induced lipotoxicity. Proc. Natl. Acad. Sci. USA.

[14] Wang Y., Qian Y., Fang Q., Zhong P., Li W., Wang L., Fu W., Zhang Y., Xu Z., Li X. (2017). Saturated palmitic acid induces myocardial inflammatory injuries through direct binding to TLR4 accessory protein MD2. Nat. Commun..

[15] Yarmohammadi F., Hayes A.W., Karimi G. (2022). Natural and chemical compounds as protective agents against cardiac lipotoxicity. Biomed. Pharmacother..

[16] Alam W., Rocca C., Khan H., Hussain Y., Aschner M., De Bartolo A., Amodio N., Angelone T., Cheang W.S. (2021). Current Status and Future Perspectives on Therapeutic Potential of Apigenin: Focus on Metabolic-Syndrome-Dependent Organ Dysfunction. Antioxidants.

[17] Casacchia T., Occhiuzzi M.A., Grande F., Rizzuti B., Granieri M.C., Rocca C., Gattuso A., Garofalo A., Angelone T., Statti G. (2019). A pilot study on the nutraceutical properties of the Citrus hybrid Tacle® as a dietary source of polyphenols for supplementation in metabolic disorders. J. Funct. Foods.

[18] Casacchia T., Scavello F., Rocca C., Granieri M.C., Beretta G., Amelio D., Gelmini F., Spena A., Mazza R., Toma C.C. (2019). Leopoldia comosa prevents metabolic disorders in rats with high-fat diet-induced obesity. Eur. J. Nutr..

[19] Nettore I.C., Rocca C., Mancino G., Albano L., Amelio D., Grande F., Puoci F., Pasqua T., Desiderio S., Mazza R. (2019). Quercetin and its derivative Q2 modulate chromatin dynamics in adipogenesis and Q2 prevents obesity and metabolic disorders in rats. J. Nutr. Biochem..

[20] Li Y., Yao J., Han C., Yang J., Chaudhry M.T., Wang S., Liu H., Yin Y. (2016). Quercetin, Inflammation and Immunity. Nutrients.

[21] Yang D., Wang T., Long M., Li P. (2020). Quercetin: Its Main Pharmacological Activity and Potential Application in Clinical Medicine. Oxid. Med. Cell Longev..

[22] Jung C.H. (2013). Quercetin reduces high-fat diet-induced fat accumulation in the liver by regulating lipid metabolism genes. Phytother. Res..

[23] Panchal S.K., Poudyal H., Brown L. (2012). Quercetin Ameliorates Cardiovascular, Hepatic, and Metabolic Changes in Diet-Induced Metabolic Syndrome in Rats. J. Nutr..

[24] Papakyriakopoulou P., Velidakis N., Khattab E., Valsami G., Korakianitis I., Kadoglou N.P. (2022). Potential Pharmaceutical Applications of Quercetin in Cardiovascular Diseases. Pharmaceuticals.

[25] Grande F., Parisi O.I., Mordocco R.A., Rocca C., Puoci F., Scrivano L., Quintieri A.M., Cantafio P., Ferla S., Brancale A. (2016). Quercetin derivatives as novel antihypertensive agents: Synthesis and physiological characterization. Eur. J. Pharm. Sci..

[26] Iacopetta D., Grande F., Caruso A., Mordocco R.A., Plutino M.R., Scrivano L., Ceramella J., Muià N., Saturnino C., Puoci F. (2017). New insights for the use of quercetin analogs in cancer treatment. Future Med. Chem..

[27] Rocca C., De Bartolo A., Grande F., Rizzuti B., Pasqua T., Giordano F., Granieri M.C., Occhiuzzi M.A., Garofalo A., Amodio N. (2021). Cateslytin abrogates lipopolysaccharide-induced cardiomyocyte injury by reducing inflammation and oxidative stress through toll like receptor 4 interaction. Int. Immunopharmacol..

[28] Rocca C., Grande F., Granieri M.C., Colombo B., De Bartolo A., Giordano F., Rago V., Amodio N., Tota B., Cerra M.C. (2021). The chromogranin A1-373 fragment reveals how a single change in the protein sequence exerts strong cardioregulatory effects by engaging neuropilin-1. Acta Physiol. (Oxf)..

[29] Grande F., De Bartolo A., Occhiuzzi M.A., Caruso A., Rocca C., Pasqua T., Carocci A., Rago V., Angelone T., Sinicropi M.S. (2021). Carbazole and Simplified Derivatives: Novel Tools toward β-Adrenergic Receptors Targeting. Appl. Sci..

[30] Rocca C., De Bartolo A., Granieri M.C., Rago V., Amelio D., Falbo F., Malivindi R., Mazza R., Cerra M.C., Boukhzar L. (2022). The Antioxidant Selenoprotein T Mimetic, PSELT, Induces Preconditioning-like Myocardial Protection by Relieving Endoplasmic-Reticulum Stress. Antioxidants.

[31] Dong Q., Chen L., Lu Q., Sharma S., Li L., Morimoto S., Wang G. (2014). Quercetin attenuates doxorubicin cardiotoxicity by modulating Bmi-1 expression. Br. J. Pharmacol..

[32] McQueen M.J. (1972). Optimal Assay of LDH and α-HBD at 37 °C. Ann. Clin. Biochem..

[33] Rocca C., Scavello F., Colombo B., Gasparri A.M., Dallatomasina A., Granieri M.C., Amelio D., Pasqua T., Cerra M.C., Tota B. (2019). Physiological levels of chromogranin A prevent doxorubicin-induced cardiotoxicity without impairing its anticancer activity. FASEB J..

[34] Ivan A., Herman H., Balta C., Hadaruga D.I., Mihali C.V., Ardelean A., Hermenean A. (2017). Berberis vulgaris extract/β-cyclodextrin complex increases protection of hepatic cells via suppression of apoptosis and lipogenesis pathways. Exp. Ther. Med..

[35] Shen C.J., Kong B., Shuai W., Liu Y., Wang G.J., Xu M., Zhao J.J., Fang J., Fu H., Jiang X.B. (2019). Myeloid differentiation protein 1 protected myocardial function against high-fat stimulation induced pathological remodelling. J. Cell Mol. Med..

[36] Nasci V.L., Chuppa S., Griswold L., Goodreau K.A., Dash R.K., Kriegel A.J. (2019). miR-21-5p regulates mitochondrial respiration and lipid content in H9C2 cells. Am. J. Physiol. Heart Circ. Physiol..

[37] Assimakopoulos S.F., Vagianos C.E., Zervoudakis G., Filos K.S., Georgiou C., Nikolopoulou V., Scopa C.D. (2004). Gut regulatory peptides bombesin and neurotensin reduce hepatic oxidative stress and histological alterations in bile duct ligated rats. Regul. Pept..

[38] Preetha Rani M.R., Anupama N., Sreelekshmi M., Raghu K.G. (2018). Chlorogenic acid attenuates glucotoxicity in H9c2 cells via inhibition of glycation and PKC α upregulation and safeguarding innate antioxidant status. Biomed. Pharmacother..

[39] Reznick A.Z., Packer L. (1994). Oxidative damage to proteins: Spectrophotometric method for carbonyl assay. Methods Enzymol..

[40] Pasqua T., Rocca C., Lupi F.R., Baldino N., Amelio D., Parisi O.I., Granieri M.C., De Bartolo A., Lauria A., Dattilo M. (2020). Cardiac and Metabolic Impact of Functional Foods with Antioxidant Properties Based on Whey Derived Proteins Enriched with Hemp Seed Oil. Antioxidants.

[41] Marklund S., Marklund G. (1974). Involvement of the superoxide anion radical in the autoxidation of pyrogallol and a convenient assay for superoxide dismutase. Eur. J. Biochem..

[42] Aebi H. (1984). Catalase in vitro. Methods Enzymol..

[43] Park E.J., Lee A.Y., Park S., Kim J.H., Cho M.H. (2014). Multiple pathways are involved in palmitic acid-induced toxicity. Food Chem. Toxicol..

[44] Severson D.L. (2004). Diabetic cardiomyopathy: Recent evidence from mouse models of type 1 and type 2 diabetes. Can. J. Physiol. Pharmacol..

[45] van den Brom C.E., Huisman M.C., Vlasblom R., Boontje N.M., Duijst S., Lubberink M., Molthoff C.F., Lammertsma A.A., van der Velden J., Boer C. (2009). Altered myocardial substrate metabolism is associated with myocardial dysfunction in early diabetic cardiomyopathy in rats: Studies using positron emission tomography. Cardiovasc. Diabetol..

[46] Grundy S.M., Benjamin I.J., Burke G.L., Chait A., Eckel R.H., Howard B.V., Mitch W., Smith S.C., Sowers J.R. (1999). Diabetes and cardiovascular disease: A statement for healthcare professionals from the American Heart Association. Circulation.

[47] Nichols G.A., Gullion C.M., Koro C.E., Ephross S.A., Brown J.B. (2004). The incidence of congestive heart failure in type 2 diabetes: An update. Diabetes Care.

[48] Wende A.R., Abel E.D. (2010). Lipotoxicity in the heart. Biochim. Biophys. Acta.

[49] Kuwabara Y., Horie T., Baba O., Watanabe S., Nishiga M., Usami S., Izuhara M., Nakao T., Nishino T., Otsu K. (2015). MicroRNA-451 exacerbates lipotoxicity in cardiac myocytes and high-fat diet-induced cardiac hypertrophy in mice through suppression of the LKB1/AMPK pathway. Circ. Res..

[50] Urso C.J., Zhou H. (2021). Role of CD36 in Palmitic Acid Lipotoxicity in Neuro-2a Neuroblastoma Cells. Biomolecules.

[51] Hescheler J., Meyer R., Plant S., Krautwurst D., Rosenthal W., Schultz G. (1991). Morphological, biochemical, and electrophysiological characterization of a clonal cell (H9c2) line from rat heart. Circ. Res..

[52] Yang Z., Chen Y., Yan Z., Xu T.T., Wu X., Pi A., Liu Q., Chai H., Li S., Dou X. (2021). Inhibition of TLR4/MAPKs Pathway Contributes to the Protection of Salvianolic Acid Against Lipotoxicity-Induced Myocardial Damage in Cardiomyocytes and Obese Mice. Front. Pharmacol..

[53] Kong J.Y., Rabkin S.W. (2000). Palmitate-induced apoptosis in cardiomyocytes is mediated through alterations in mitochondria: Prevention by cyclosporin A. Biochim. Biophys. Acta.

[54] Leroy C., Tricot S., Lacour B., Grynberg A. (2008). Protective effect of eicosapentaenoic acid on palmitate-induced apoptosis in neonatal cardiomyocytes. Biochim. Biophys. Acta.

[55] Di Majo D., Sardo P., Giglia G., Di Liberto V., Zummo F.P., Zizzo M.G., Caldara G.F., Rappa F., Intili G., van Dijk R.M. (2023). Correlation of Metabolic Syndrome with Redox Homeostasis Biomarkers: Evidence from High-Fat Diet Model in Wistar Rats. Antioxidants.

[56] Chen X., Peng X., Luo Y., You J., Yin D., Xu Q., He H., He M. (2019). Quercetin protects cardiomyocytes against doxorubicin-induced toxicity by suppressing oxidative stress and improving mitochondrial function via 14-3-3γ. Toxicol. Mech. Methods.

[57] Abushouk A.I., Ismail A., Salem A.M.A., Afifi A.M., Abdel-Daim M.M. (2017). Cardioprotective mechanisms of phytochemicals against doxorubicin-induced cardiotoxicity. Biomed. Pharmacother..

[58] Wang S.Q., Zhu X.F., Wang X.N., Shen T., Xiang F., Lou H.X. (2013). Flavonoids from Malus hupehensis and their cardioprotective effects against doxorubicin-induced toxicity in H9c2 cells. Phytochemistry.

[59] Ojha S., Al Taee H., Goyal S., Mahajan U.B., Patil C.R., Arya D.S., Rajesh M. (2016). Cardioprotective Potentials of Plant-Derived Small Molecules against Doxorubicin Associated Cardiotoxicity. Oxid. Med. Cell Longev..

[60] Chen Y.W., Chou H.C., Lin S.T., Chen Y.H., Chang Y.J., Chen L., Chan H.L. (2013). Cardioprotective Effects of Quercetin in Cardiomyocyte under Ischemia/Reperfusion Injury. Evid Based Complement Alternat Med..

[61] Li S., Zhang H., Chen K., Jin M., Vu S.H., Jung S., He N., Zheng Z., Lee M.S. (2022). Application of chitosan/alginate nanoparticle in oral drug delivery systems: Prospects and challenges. Drug Deliv..

[62] Chang X., Zhang T., Meng Q., Wang S., Yan P., Wang X., Luo D., Zhou X., Ji R. (2021). Quercetin Improves Cardiomyocyte Vulnerability to Hypoxia by Regulating SIRT1/TMBIM6-Related Mitophagy and Endoplasmic Reticulum Stress. Oxid. Med. Cell Longev..

[63] Jain A.K., Mehra N.K., Swarnakar N.K. (2015). Role of Antioxidants for the Treatment of Cardiovascular Diseases: Challenges and Opportunities. Curr. Pharm. Des..

[64] Chimento A., Sala M., Gomez-Monterrey I.M., Musella S., Bertamino A., Caruso A., Sinicropi M.S., Sirianni R., Puoci F., Parisi O.I. (2013). Biological activity of 3-chloro-azetidin-2-one derivatives having interesting antiproliferative activity on human breast cancer cell lines. Bioorg. Med. Chem. Lett..

[65] Kim M.K., Park K.S., Lee C., Park H.R., Choo H., Chong Y. (2010). Enhanced stability and intracellular accumulation of quercetin by protection of the chemically or metabolically susceptible hydroxyl groups with a pivaloxymethyl (POM) promoiety. J. Med. Chem..

[66] Kandemir K., Tomas M., McClements D.J., Capanoglu E. (2022). Recent advances on the improvement of quercetin bioavailability. Trends Food Sci. Technol..

[67] Wei C.D., Li Y., Zheng H.Y., Tong Y.Q., Dai W. (2013). Palmitate induces H9c2 cell apoptosis by increasing reactive oxygen species generation and activation of the ERK1/2 signaling pathway. Mol. Med. Rep..

[68] Wei C.D., Li Y., Zheng H.Y., Sun K.S., Tong Y.Q., Dai W., Wu W., Bao A.Y. (2012). Globular adiponectin protects H9c2 cells from palmitate-induced apoptosis via Akt and ERK1/2 signaling pathways. Lipids Health Dis..

[69] Zou L., Li X., Wu N., Jia P., Liu C., Jia D. (2017). Palmitate induces myocardial lipotoxic injury via the endoplasmic reticulum stress-mediated apoptosis pathway. Mol. Med. Rep..

[70] Cai L., Wang Y., Zhou G., Chen T., Song Y., Li X., Kang Y.J. (2006). Attenuation by metallothionein of early cardiac cell death via suppression of mitochondrial oxidative stress results in a prevention of diabetic cardiomyopathy. J. Am. Coll. Cardiol..

[71] Cai L. (2006). Suppression of nitrative damage by metallothionein in diabetic heart contributes to the prevention of cardiomyopathy. Free Radic. Biol. Med..

[72] Li Y.J., Wang P.H., Chen C., Zou M.H., Wang D.W. (2010). Improvement of mechanical heart function by trimetazidine in db/db mice. Acta Pharmacol. Sin..

[73] He L., He T., Farrar S., Ji L., Liu T., Ma X. (2017). Antioxidants Maintain Cellular Redox Homeostasis by Elimination of Reactive Oxygen Species. Cell Physiol. Biochem..

[74] Rocca C., Pasqua T., Boukhzar L., Anouar Y., Angelone T. (2019). Progress in the emerging role of selenoproteins in cardiovascular disease: Focus on endoplasmic reticulum-resident selenoproteins. Cell Mol. Life Sci..

[75] Wu K.M., Hsu Y.M., Ying M.C., Tsai F.J., Tsai C.H., Chung J.G., Yang J.S., Tang C.H., Cheng L.Y., Su P.H. (2019). High-density lipoprotein ameliorates palmitic acid-induced lipotoxicity and oxidative dysfunction in H9c2 cardiomyoblast cells via ROS suppression. Nutr. Metab..

[76] Qian P., Tian H., Wang Y., Lu W., Li Y., Ma T., Gao X., Yao W. (2020). A novel oral glucagon-like peptide 1 receptor agonist protects against diabetic cardiomyopathy via alleviating cardiac lipotoxicity induced mitochondria dysfunction. Biochem. Pharmacol..

[77] Kurutas E.B. (2016). The importance of antioxidants which play the role in cellular response against oxidative/nitrosative stress: Current state. Nutr. J..

[78] Roslan J., Giribabu N., Karim K., Salleh N. (2017). Quercetin ameliorates oxidative stress, inflammation and apoptosis in the heart of streptozotocin-nicotinamide-induced adult male diabetic rats. Biomed. Pharmacother..

[79] Yu S., Kim S.R., Jiang K., Ogrodnik M., Zhu X.Y., Ferguson C.M., Tchkonia T., Lerman A., Kirkland J.L., Lerman L.O. (2021). Quercetin Reverses Cardiac Systolic Dysfunction in Mice Fed with a High-Fat Diet: Role of Angiogenesis. Oxid. Med. Cell Longev..

[80] Doroshow J.H., Locker G.Y., Myers C.E. (1980). Enzymatic defenses of the mouse heart against reactive oxygen metabolites: Alterations produced by doxorubicin. J. Clin. Investig..

[81] Rocca C., Pasqua T., Cerra M.C., Angelone T. (2020). Cardiac Damage in Anthracyclines Therapy: Focus on Oxidative Stress and Inflammation. Antioxid. Redox Signal..

[82] Nishi H., Higashihara T., Inagi R. (2019). Lipotoxicity in Kidney, Heart, and Skeletal Muscle Dysfunction. Nutrients.

[83] Warbrick I., Rabkin S.W. (2019). Hypoxia-inducible factor 1-alpha (HIF-1α) as a factor mediating the relationship between obesity and heart failure with preserved ejection fraction. Obes. Rev..

[84] Fillmore N., Mori J., Lopaschuk G.D. (2014). Mitochondrial fatty acid oxidation alterations in heart failure, ischaemic heart disease and diabetic cardiomyopathy. Br. J. Pharmacol..

[85] Mangali S., Bhat A., Udumula M.P., Dhar I., Sriram D., Dhar A. (2019). Inhibition of protein kinase R protects against palmitic acid-induced inflammation, oxidative stress, and apoptosis through the JNK/NF-kB/NLRP3 pathway in cultured H9C2 cardiomyocytes. J. Cell Biochem..

[86] Zhang K., Kaufman R.J. (2008). From endoplasmic-reticulum stress to the inflammatory response. Nature.

[87] Glass C.K., Olefsky J.M. (2012). Inflammation and lipid signaling in the etiology of insulin resistance. Cell Metab..

[88] Maloney E., Sweet I.R., Hockenbery D.M., Pham M., Rizzo N.O., Tateya S., Handa P., Schwartz M.W., Kim F. (2009). Activation of NF-kappaB by palmitate in endothelial cells: A key role for NADPH oxidase-derived superoxide in response to TLR4 activation. Arterioscler. Thromb. Vasc. Biol..

[89] Niebauer J. (2000). Inflammatory mediators in heart failure. Int. J. Cardiol..

[90] Shan K., Kurrelmeyer K., Seta Y., Wang F., Dibbs Z., Deswal A., Lee-Jackson D., Mann D.L. (1997). The role of cytokines in disease progression in heart failure. Curr. Opin. Cardiol..

[91] Sharma R., Coats A.J., Anker S.D. (2000). The role of inflammatory mediators in chronic heart failure: Cytokines, nitric oxide, and endothelin-1. Int. J. Cardiol..

[92] Forney L.A., Lenard N.R., Stewart L.K., Henagan T.M. (2018). Dietary Quercetin Attenuates Adipose Tissue Expansion and Inflammation and Alters Adipocyte Morphology in a Tissue-Specific Manner. Int. J. Mol. Sci..

[93] Sato S., Mukai Y. (2020). Modulation of Chronic Inflammation by Quercetin: The Beneficial Effects on Obesity. J. Inflamm. Res..

